# Cancer Stem Cells in Soft-Tissue Sarcomas

**DOI:** 10.3390/cells9061449

**Published:** 2020-06-10

**Authors:** Paula Martínez-Delgado, Serena Lacerenza, Antonia Obrador-Hevia, Maria Lopez-Alvarez, José L. Mondaza-Hernandez, Elena Blanco-Alcaina, Paloma Sanchez-Bustos, Nadia Hindi, David S. Moura, Javier Martin-Broto

**Affiliations:** 1 Institute of Biomedicine of Seville (IBIS, HUVR, CSIC, Universidad de Sevilla), 41013 Sevilla, Spain; paula.mrtnez.delgado@gmail.com (P.M.-D.); serelac@hotmail.it (S.L.); marlopalv4@gmail.com (M.L.-A.); joseluciniomondaza@gmail.com (J.L.M.-H.); elena.blancoalcaina@gmail.com (E.B.-A.); sanchezbustospaloma@gmail.com (P.S.-B.); nhindi@mustbesevilla.org (N.H.); david.moura@usal.es (D.S.M.); 2 Health Research Institute of the Balearic Islands (IdISBa) and Molecular Diagnosis Unit, Son Espases University Hospital, 07120 Palma de Mallorca, Spain; antonia.obrador@ssib.es; 3 Medical Oncology Department, University Hospital Virgen del Rocio, 41013 Seville, Spain

**Keywords:** cancer stem cells, tumor-initiating cells, soft-tissue sarcoma, chemotherapy resistance, stemness, tumor heterogeneity, genetic and epigenetic plasticity

## Abstract

Soft tissue sarcomas (STS) are a rare group of mesenchymal solid tumors with heterogeneous genetic profiles and clinical features. Systemic chemotherapy is the backbone treatment for advanced STS; however, STS frequently acquire resistance to standard therapies, which highlights the need to improve treatments and identify novel therapeutic targets. Increases in the knowledge of the molecular pathways that drive sarcomas have brought to light different molecular alterations that cause tumor initiation and progression. These findings have triggered a breakthrough of targeted therapies that are being assessed in clinical trials. Cancer stem cells (CSCs) exhibit mesenchymal stem cell (MSC) features and represent a subpopulation of tumor cells that play an important role in tumor progression, chemotherapy resistance, recurrence and metastasis. In fact, CSCs phenotypes have been identified in sarcomas, allied to drug resistance and tumorigenesis. Herein, we will review the published evidence of CSCs in STS, discussing the molecular characteristic of CSCs, the commonly used isolation techniques and the new possibilities of targeting CSCs as a way to improve STS treatment and consequently patient outcome.

## 1. Introduction

Cancer stem cells (CSCs), also known as tumor-initiating cells (TICs), are cancer cells that display distinctive features of normal stem cells and that have the ability to give origin to all cell types of a particular cancer sample. Moreover, these cells seem to arise due to the deregulated control of core pluripotency factors and they have been linked to chemotherapy resistance in sarcomas. CSCs can originate from (1) mutations in stem cells; (2) somatic differentiated cells with high rate of cell turnover by genomic instability, horizontal gene transfer or microenvironment changes; (3) cell fusion; and (4) metabolic reprograming [1]. However, frequently CSC subpopulations emerge after the accumulation of epigenetic and/or genetic changes in a cell within the aberrant population, initially generated by the cell of origin, namely, mesenchymal stem cell (MSC)-derived cell types [2]. It should be noted that the cell of origin, in which tumorigenesis is initiated, may be distinct from the CSC, which propagates the tumor [3]. Sustaining their expected mesenchymal progenitor origin, many of these sarcoma-initiating cells express MSC markers and keep in vitro differentiation properties [2]. The relationship between MSCs and tumor microenvironment (TME) of CSCs is essential to maintain Cancer Stemness.

Likewise, subpopulations of CSCs seem to drive the evolution of tumors from initial carcinogenic events into increasingly aggressive neoplasias. In sarcomas, CSCs presenting MSC features have been identified and linked to tumor initiation and the mechanisms of relapse and metastasis [4,5,6].

Doxorubicin is the backbone for the first-line treatment of metastatic Soft-Tissue Sarcomas (STS). The combination of doxorubicin with other drugs, such as Olaratumab, palifosfamide or evofosfamide, among others have been evaluated in several comparative clinical trials against doxorubicin; however, no significant survival improvement was detected when compared with the single agent [7,8,9]. In spite of initial drug activity, patients finally progress in the majority of the cases. Thus, the failure of these doxorubicin combinations could be in part due to the acquisition of multidrug resistance (MDR) mechanisms [10]. Nevertheless, more translational studies are needed to address the molecular basis and clinical relevance of MDR, which seems to be related with the existence of CSCs. In fact, CSCs have been described to be involved in drug resistance through various mechanisms, including activation of resting/quiescent cells, elevation of drug efflux pumps and elevated drug detoxification, efficient activation of DNA damage repair and resistance to apoptosis. Thus, the disruption of such mechanisms could improve sarcoma patient prognosis [1]. Likewise, a better understanding of the molecular characteristics of CSCs may help the development of specific sarcoma stem cell-targeted therapies, which could finally improve patient outcome. In the sections below, we will review the molecular characteristics of CSCs and how these features could be used to target CSCs in STS.

## 2. Isolation and Molecular Characterization of Cancer Stem Cells (CSCs) in Soft Tissue Sarcomas (STS)

From a molecular perspective, CSCs are considerably different from the other tumor cells. In the 1990s, CSCs were identified for the first time in leukemia as CD34+ and CD38- cells with high proliferative rate and tumor-forming capacities in vivo [11]. Generally, CSCs share some common markers with normal stem cells, but stem cell markers in CSCs appear to be upregulated, or mutations have been described [12]. Also, common stem-cell-associated pathways have been shown to be aberrantly expressed in CSCs [13]. Thereby, the pathways that regulate the balance between self-renewal and differentiation in normal stem cells are rather uncontrolled in CSCs, leading to tumorigenesis. 

CSCs expressing markers such as CD133, nestin, and CD44, have been found in many tumors [14]. Thus, CSC subpopulations have been detected and isolated primarily via the immunodetection of CSC markers on the cell surface using sorting methods, such as the fluorescence-activated cell sorting (FACS) or the magnetic-activated cell sorting (MACS), and also through CSC-related functional features, like the expression/over-expression of protective cellular enzymes and membrane transporters [15]. In vitro assays have been used to identify CSC functional characteristics: assessment of ALDH activity, “sarcosphere” formation assays, clonogenicity assays, and detection of a side population. However, the gold standard for the detection of CSCs are the in vivo tumorigenicity assays in immunodeficient mice, where CSCs self-renewal and tumor-forming capacities are directly tested in a living organism [16,17].

**Figure 1:**
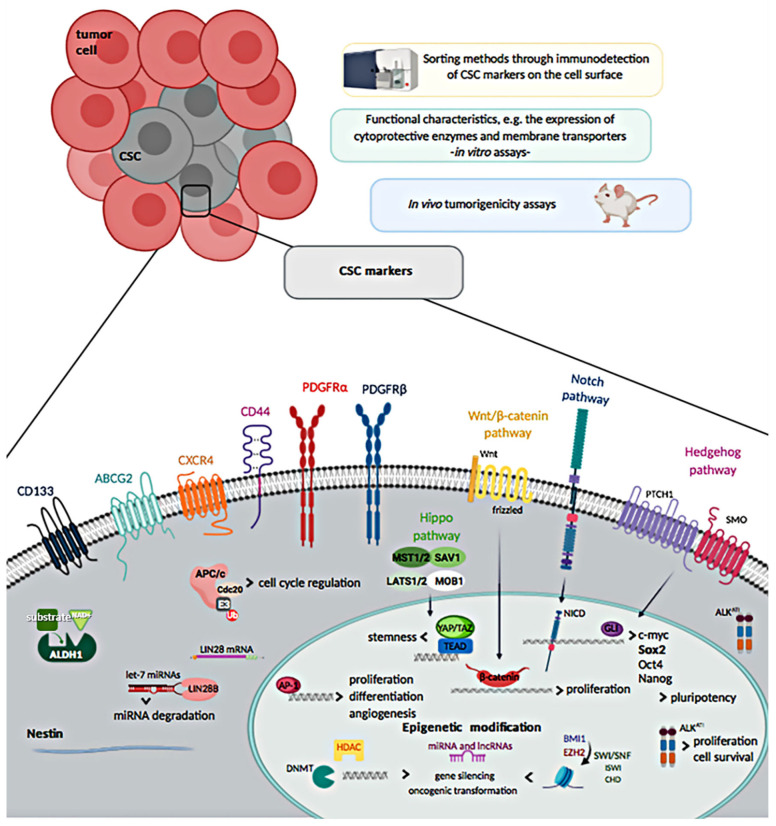
Methods to isolate cancer stem cells (CSCs) and overview of soft-tissue sarcoma CSCs markers.

Of note, several surface markers that over time have been described to be expressed by cells showing distinctive features of CSCs, such as high proliferative rate, high clonogenicity and tumorigenicity, have been also used with the purpose of identifying them among the heterogeneous cell populations of STS, and also as outcome predictors. Nevertheless, their role and use for detection of CSCs in STS remains controversial, due to the fact that many CSC markers could also define normal MSCs [4]. The different approaches used to discriminate CSCs in STS are detailed in the following sections and shown in Figure 1.

### 2.1. Cell Surface Markers

#### 2.1.1. CD133

CD133, also known as prominin-1, is a membrane protein considered as putative CSC marker in various cancers, including sarcomas, at least in early studies. In fact, CD133 was the first protein reported to identify CSCs in Ewing’s sarcoma (EWS) [18] or rhabdomyosarcoma (RMS) [19,20,21]. In vitro studies in sarcoma cell lines have reported that CD133+ cells mark a subpopulation of cancer cells characterized by high expression levels of stemness genes, in vitro self-renewal, and increased clonogenicity and tumorigenicity [19,20,22,23,24,25]. However, contradictory results related to the specificity value of CD133 as a marker of sarcoma CSCs have been published in pediatric sarcomas [26].

Noteworthy, CD133 expression has been correlated with poor survival in embryonal rhabdomyosarcoma (ERMS) [20]. Similar results were reported in pediatric sarcomas, where high expression of CD133 and nestin, but not of ABCG2, were associated with shorter survival in RMS and EWS patients, supporting the potential prognostic value of CD133 in sarcomas [27].

#### 2.1.2. Chemokine Receptor Type-4

Chemokine receptor type-4 (CXCR4) has been described to be overexpressed in synovial sarcoma (SS), where the CXCR4+ cells have been outlined to have a higher tumor initiation potential in in vivo experiments and a higher sphere formation capacity. Noteworthy, immunohistochemistry (IHC) analysis of SS patients’ specimens revealed a strong correlation between CXCR4 expression and poor prognosis [28]. It has been described that CXCR4 expression can be affected by microRNAs (miRNAs), a class of 21–23 nucleotides RNA molecules that regulate in a large-scale gene expression and play a crucial role in mediating CSCs features. Remarkably, the overexpression of the miRNA miR-494.3p downregulated *CXCR4* gene and protein expression levels, leading to a transitory decrease in SS cell proliferation and migration. However, no effect on CSCs was reported in this study [29]. CXCR4 overexpression was described to be associated with worse 5-year overall survival linked to the use of chemotherapy (47% vs 86%), in a series of 88 SS. Further studies should address whether CXCR4 is a potential CSC marker specific for SS or for a broad spectrum of STS subtypes [30].

#### 2.1.3. CD44

CD44 has been used to identify CSCs sub-populations in pre-clinical and clinical models of many tumors. Yet, its potential value to discriminate CSCs sub-populations in STS is very limited and it has been reported that this cell surface marker could not be a useful tool to isolate CSCs in STS [31]. Nonetheless, elevated levels of CD44 expression were associated with worse outcome in STS; however, and since CD44 has pleotropic cellular functions, this association might be unrelated to the stem-like behavior of CD44+ STS cells [32].

#### 2.1.4. Nestin

Nestin is a neuronal cytoplasmic stem cell protein that belongs to class VI intermediate filaments. Functionally, it is linked with normal stem cells and has been extensively studied as CSCs marker. Nestin has been shown to be expressed in different cells and tissues and in many types of cancer, including in specific sarcoma subtypes, such as RMS and malignant peripheral nerve sheath tumor (MPNST) [33,34,35,36]. Consequently, it has been suggested that nestin could be a putative CSC marker for tumors of mesenchymal origin. In addition, it has been described that the co-expression of nestin with other CSC markers, especially CD133, in RMS patient samples and cell lines, can be regarded as a specific CSC phenotype [19]. Cells positive for those markers have a higher tumorigenic potential; however, further functional validations are required to confirm these observations, and to verify if these CSCs marker profiles can be used for other STS subtypes [34]. On the other hand, high expression of nestin has been correlated with poor prognosis in EWS, suggesting that this protein may also be a prognostic factor in sarcomas [27].

#### 2.1.5. ALDH1

ALDHs belong to a family of enzymes which are responsible for detoxifying endogenous and exogenous aldehydes to carboxylic acids through NAD(P)+-dependent oxidation. Normal stem cells show high ALDH activity, thus it is considered a universal marker of stemness, as well as of CSCs [37]. Subpopulations of STS cell lines with increased ALDH activity were shown to have higher proliferation and clonogenicity. This has been reported in EWS [38], ERMS [39], SS and fibrosarcoma [40]. Likewise, a small fraction of cells (0.1–1.7%) characterized by a stem-like phenotype (CD133+ and ALDH+) has been identified in a liposarcoma xenograft model. This putative CSC population showed in vitro self-renewal ability, capacity to differentiate into mature adipocytes and in vivo strong tumorigenicity [41]. Of note, in sarcomas CSC phenotype is associated only with increased ALDH activity, but not with the most common ALDH1 isoform expression [26]. ALHD1, CD133, and CD44 are among the most investigated CSC markers; however, their value as CSCs markers in STS needs further confirmations. Very recently, the expression of these CSCs markers before and after pre-operative chemotherapy was examined in STS samples of patients enrolled on a prospective clinical trial. No significant changes on the protein levels of these markers, between pre- and post-treatment STS samples, was detected by IHC, suggesting that these proteins may not be altered by treatment. Importantly, tumor-infiltrating macrophages, often prominent in the tumor environment, also expressed CD44 and ALDH1. Thus, detection of CSCs with these latter markers was not straightforward. Overall, ALDH1, CD133, and CD44 have been proved to be valuable in identifying CSCs in epithelial tumors, while their usefulness as CSC markers in STS is limited [31].

#### 2.1.6. PDGFRα and PDGFRβ

The role of platelet-derived growth factor receptors α and β (PDGFR-α/β) have been recently examined in sarcoma CSCs, as they are known to be important regulators of MSC growth and differentiation [42,43]. The results obtained in fibrosarcoma, leiomyosarcoma and liposarcoma cell lines and mouse xenograft models, indicated that in sarcoma CSCs PDGFR-α/β activity is upregulated and promoted CSC phenotype, including cell migration and invasion, and chemotherapy resistance. This CSC phenotype was inhibited by the downregulation of PDGFR-α/β levels [44]. Nonetheless, the inhibition of PDGFRα with olaratumab (a human IgG1 antibody targeting PDGFRα) did not showed an enhancement of chemotherapy activity in advanced STS; the phase 3 clinical trial testing the combination of olaratumab and doxorubicin versus doxorubicin did not meet its primary endpoint [7]. Correlative studies associated with this trial are expected, in order to understand if PDGFRβ signaling pathway could have a role in tumor progression after PDGFRα inhibition and to identify those patients who could benefit from doxorubicin plus olaratumab combination. Noteworthy, dermatofibrosarcoma protuberans (DFSP), a rare STS subtype with a local infiltrative growth pattern, carries a *COL1A1/PDGFB* fusion-gene that is responsible for PDGFRβ autocrine activation and subsequent tumorigenesis [45]. Classic DFSP are chemoresistant.However, PDGFRβ inhibition with imatinib has shown efficacy in DFSP [46], and further investigations are warranted to understand the mechanisms underlying imatinib-resistance in a subset of DFSP patients [47].

#### 2.1.7. ATP-Binding Cassette (ABC) Efflux Pumps

The ABC family of efflux pumps are membrane-associated proteins whose function is to transport various molecules across extra- and intra-cellular membranes of numerous cell types. ABC transporters constitute the principle mechanism of protection for stem cells. Even this feature has been extensively described to be shared by CSCs and to protect them from the effects of chemotherapy [48]. Moreover, it has been reported that ABC transporters, especially MRP-1 (*ABCC1*), ABCG2 and P-gp (*ABCB1*), are associated with worse prognosis in sarcomas, since these pumps mediate multidrug resistance mechanisms [49,50]. ABC transporters define the Side Population (SP) [48], which is a sub-population of CSCs that drives cancer progression. SP will be further described in Section 2.2. High expression of ABCG2 was detected in CSCs isolated from undifferentiated pleomorphic sarcoma (UPS) cell line. These cells were described to have enhanced self-renewal capacity and a remarkable doxorubicin and cisplatin resistance. Increased efficacy of the aforesaid chemotherapeutic agents was reached when administered with an ABCG2 inhibitor [51].

Overall, none of these CSC markers described above seem to unequivocally identify CSCs in STS, and more likely they might be sarcoma-subtype specific [5]. Therefore, the combined use of some of these markers with other approaches, which rely on some of the CSCs characteristics, could be useful to better isolate and delineate the CSC phenotype in STS.

### 2.2. Side Population

It has been pointed out that CSCs harbor tumorigenic SP [52], an observation that is in line with the hierarchical model of CSC, which suggests that a sub-population of CSCs has the ability to drive tumor progression. The SP phenotype is characterized by higher drug efflux capability, higher ability to transdifferentiate and to sustain expansion, and high tumorigenicity in immunocompromised mice [53]. These characteristics are established criteria for CSCs. Hence, SP have been used to enrich for CSCs in several types of cancer, including STS [54]. Recently, it was shown that EWS SP cells only shared a limited number of MSC genes and had different characteristics compared with the core cell population. In this study, activator protein 1 (AP-1)-mediated gene transcription and Anaphase Promoting Complex cyclosome (APC/c)-CDC20-mediated cell cycle regulation seemed to underlie SP CSC properties. APC/c-CDC20 is a complex formed by the APC/c ubiquitin ligase enzyme, and the oncogene *CDC20*. Moreover, the histone deacetylase HDAC9 was described to be the main epigenetic modifier supporting SP stemness features [55]. In a study with 29 mesenchymal tumors, processed at the time of surgical excision, SP was found in samples of SS, leiomyosarcoma, UPS, myxoid liposarcoma and pleomorphic liposarcoma, as well as in aggressive fibromatosis, osteosarcoma, chondrosarcoma and chordoma [56].

### 2.3. Stemness Markers

Epigenetic and genetic changes involved in the sarcomagenesis have a deep impact on the biology of sarcoma stem cells. Certain subtypes of sarcoma are characterized by stable karyotypes and reciprocal translocations, leading to activated fusion-oncogenes that can be crucial in conferring stemness, as they have the ability to modify the transcriptome and to interfere with normal epigenetic processes, as shown for EWS and SS [57]. For sarcomas featuring unstable karyotypes and high somatic mutations, stemness derives from the assembly of epigenetically and genetically conditioned stemness factors [57]. Otherwise, stemness can be enforced by TME, as widely described in osteosarcoma. Notwithstanding this heterogeneity, diverse sarcoma types share similar molecular mechanisms of stemness, such as the abrogation of classical tumor suppressors p53 and Rb, Sox2 activation, or inhibition of canonical Wnt/β-catenin signaling pathway. Even some stem cell markers initially characterized in various carcinomas or leukemias seem to have similar stem cell enrichment capacity in sarcomas [4]. Moreover, CSCs have been described to express developmental pathways such as Wnt/β-catenin, Notch, Hedgehog and Hippo; they are key evolutionarily conserved signaling pathways involved in the regulation of stemness and differentiation. Active Hedgehog and Notch pathways have been mainly observed in ERMS and UPS CSCs [58,59,60,61], while increased expression of YAP/TAZ, components of the Hippo pathways, were correlated with the acquisition of CSC traits and maintenance of stem cell plasticity in ERMS [62].

Besides, CSCs express embryonic transcription factors (TFs) such as Sox2, Nanog, Oct4, Klf4, and Myc, which form a transcriptional network in which they mutually co-regulate each other, control chromatin remodeling and modulate gene expression patterns. Remarkably, high expression levels of Sox2 are indispensable for sarcoma cell lines to form tumors in immunodeficient mice [26], which suggests that the expression of this TF correlates with a CSC phenotype. Additionally, it has been highlighted that all the sarcoma cell lines with high ALDH activity overexpress Sox2 [40]. In normal stem cells, Sox2, together with Oct4 and Nanog, are referred as the key embryonic stem cell (ESC) factors sustaining pluripotency. Nevertheless, it has been also demonstrated that these TFs induce and maintain CSCs in many cancers [63]. A positive correlation was reported in RMS between putative CSCs and high expression of all three ESC pluripotency factors [64]. In subsequent studies, Sox2 turned out to be crucial in the maintenance of sarcoma CSCs; this TF is being considered as the major marker of CSCs in sarcomas [5].

In addition, other CSC-related genes have been described recently in sarcomas. For example, LIN28B has been identified, using next-generation sequencing (NGS), as a CSC-related gene highly expressed in isolated clones with a higher tumorigenic potential, and as CSC-related protein expressed in 85% of osteosarcoma primary tissues. LIN28 silencing changed the metabolism of cells and induced the loss of CSC traits [65]. Moreover, in sarcoma it has been reported that high expression of LIN28B and survival correlate inversely with each other [66]. Nevertheless, the value of LIN28B to discriminate CSCs populations and its relevance is poorly studied in STS. Furthermore, a new anaplastic lymphoma kinase (ALK) transcript, ALKATI, was recently encountered in STS patients, where its expression correlated with tumor grade (*p* = 0.010) and tumor size (*p* = 0.036), and with worse PFS (*p* < 0.05). ALKATI overexpression seemed to contribute to tumorigenesis, drug resistance and maintenance of CSC-like features, mostly by upregulating the expression levels of stem cell markers such as c-Myc, ABCG2, BMI-1, and Oct4.

### 2.4. Epigenetic Alterations

In the processes of CSC formation and maintenance, alterations of the epigenetic system are considered crucial. Epigenetic processes can change gene expression without causing any modification in cellular DNA and they can affect the balance of self-renewal and pluripotency versus differentiation. In line with this, it has been reported that DNA methylation might promote a CSC phenotype, and that hyperactivation of DNA methyltransferases (DNMT) is required for CSC maintenance. In fact, it has been reported that both a CSC phonotype and the oncogenic transformation of precancerous cells are induced by local DNA hypermethylation at tumor-suppressor genes or genes associated with differentiation. In the context of STS, increased expression of DNMT1 has been described in liposarcoma, where it causes promoter methylation and subsequent downregulation of the expression of tumor suppressor miR-193b [67]. In ERMS, the depletion of DNMT3B affects tumor cells phenotypes by recovering the myogenic program, thus it appears to be important in maintaining a less differentiated phenotype [68].

Histone modifications are other epigenetic processes correlated with the CSC reprogramming. In solid cancers and hematological malignancies, epigenetic modifiers are very often altered. However, the association between CSC phenotype and histone modifications has not been extensively demonstrated in STS. Some evidences have been reported for the oncogene *BMI1*, whose high expression has been described in fractions of CD133+ SS cells [23]. Further, it has been shown that a few STS subtypes overexpress the enhancer of zeste homolog 2 (EZH2) methyltransferase, which is the catalytic subunit of the polycomb protein complex 2 (PRC2) and functions as a transcription repressor via the trimethylation of lysine 27 of histone 3 (H3K27me3). This overexpression contributes to tumor suppressor genes silencing and fosters CSC expansion promoting a self-renewal transcriptional program [17]. Hence, the epigenetic modifier EZH2 expression has been found altered in ERMS and SS, where it appears to support a less differentiated and more aggressive phenotype and cancer survival [69,70,71,72]. Aberrant epigenetic regulation and EZH2 overexpression have been also described in clear cell sarcoma of the kidney [73]. PRC1 and 2-related proteins, such as BMI1 and EZH2, are linked to c-Myc expression and create feedback regulating loops, in mechanisms involving also miRNAs. An example mentioned above is miR-494.3p, which regulates cell proliferation and migration by modulating CXCR4 expression in SS [29].

Finally, ATP-dependent chromatin remodelers, such as SWI/SNF, ISWI, and CHD, are implicated in tumor initiation, sarcomagenesis and maintenance of a stem-like phenotype in STS [17], namely in myxoid liposarcoma [74], SS [75], or RMS [76]. Table 1 summarizes the CSC markers that have been used to identify, isolate and characterize CSCs in different STS subtypes.

## 3. New Therapeutic Approaches against CSCs in STS and Future Directions

Multidrug resistance mechanisms have been described to be related with chemotherapy failure for many years, not only in STS [78]. Nevertheless, a few studies had shown that some drugs, already approved in STS, could also influence CSCs populations. For example, it has been demonstrated that trabectedin was effective in inhibiting sarcoma-initiating cells growth in models of myxoid/round cell liposarcoma and UPS by repression of genes associated with a CSC phenotype. Nevertheless, a therapeutic advantage of trabectedin treatment of in vivo xenografts models over doxorubicin treatment was not obtained [79], suggesting that new therapeutic approaches targeting both CSC and non-CSC subpopulations are urgently needed to improve chemotherapy efficacy. Of note, combination of trabectedin with campthotecin synergistically caused a strong inhibition of tumor cell growth, also observed in CSC subpopulations [79].

The role of CSCs in tumorigenesis and their potential value as therapeutic targets is the focus of many researchers in all cancer subtypes. However, due to the fact that STSs are less common and exhibit enormous cellular and molecular heterogeneity, there is a gap concerning stemness and the real therapeutic value of CSCs-targeted therapies. These emerging therapeutic approaches are defined in the following sections.

### 3.1. Inhibition of CSC-Dependent Pathways and Surface Markers

#### 3.1.1. ABC Efflux Pumps

In the pre-clinical setting, CD133+ cells showed increased resistance to standard chemotherapy than CD133- cells, which highlight CSC properties of CD133+ cells. Of note, these latter cells overexpressed ABCG2, suggesting that multidrug resistance efflux pumps may be of clinical relevance in STS. However, the growth of CD133+ cells was only in part inhibited by targeted therapy with an ABCG2 antagonist, indicating that CSCs may overcome inhibition by expressing other drug-resistant proteins such as P-gp and MRP-1 [22,80]. Of note, MRP-1 was shown to be a strong prognostic factor in patients with localized high-risk STS treated with anthracyclines-based regimens, suggesting that inhibiting the activity of this efflux pump could improve the efficacy of chemotherapy [50]. In line with this, it was reported that oleanolic and maslinic acid sensitize STS cells to doxorubicin treatment, via MRP-1 inhibition [81]. Likewise, Nilotinib was shown to have a synergistic effect with doxorubicin in in vitro models of STS, by reverting doxorubicin-induced P-gp-mediated multidrug resistance and by inhibiting MRP-1 activity [82].

Overall, the ABC transporters that are overexpressed at the surface of CSCs may represent a relevant target in STS. Currently, ABC transporter third-generation inhibitors or tyrosine kinase inhibitors (TKI) in combination with chemotherapy are being tested with the main goal to improve the efficacy of chemotherapy [6]. For example, it was reported that the TKI dasatinib, but not EGFR-directed inhibition, decreased cell viability of sarcoma initiating cells in combination with doxorubicin [83]. Moreover, total topotecan exposure was 1.7-fold higher in solid tumors patients treated with topotecan in combination with pazopanib, compared with patients treated with topotecan alone, primarily due to the fact that pazopanib inhibits ABCG2, thus increasing the absorption of orally administered topotecan [84]. This combination is currently being trialed in STS and bone sarcomas (NCT02357810). Furthermore, Nilotinib has been tested in combination with doxorubicin in a phase I/II clinical trial, sponsored by the Spanish group for research on sarcomas (GEIS). Phase I results showed that the combination is feasible and it does not add substantial toxicity in comparison with doxorubicin alone. Of note, it was reported a decreased expression of both MRP-1 and P-gp after 5 days of Nilotinib treatment [85]. The results of the phase II part are expected to be published soon.

#### 3.1.2. JAK–STAT Signaling Pathway

The JAK–STAT signaling pathway has been described to be involved in tumor development and CSC maintenance. Interestingly, myxoid liposarcoma cell lines expressed different amounts of canonical JAK–STAT transcript, suggesting that this pathway may be of clinical significance in this histologic subtype. In fact, inhibition of JAK–STAT signaling pathway with ruxolitinib (JAK1/2 inhibitor) caused a reduction in the number of cells with CSC properties, which were described to be resistant to doxorubicin treatment. The combination of doxorubicin plus ruxolitinib seemed to be synergistic, targeting both CSC and proliferating cells. Nevertheless, it is important to mention that some cells with CSC features that are resistant to doxorubicin and lack active JAK–STAT signaling persisted, which indicated that additional CSC-related pathways may be also active in MLS [74]. Further studies should explore the role of these additional pathways and corroborate the clinical application of JAK–STAT inhibitors.

#### 3.1.3. DNA Damage Repair Pathways

Chemotherapeutic agents generally induce DNA damage, leading to cell apoptosis when DNA damage accumulation is critical or the DNA repair mechanisms are inefficient. The efficient DNA mismatch repair that is characteristic of CSCs seems to be also important in the mechanisms of drug resistance. Indeed, it was described that the resistance to both cisplatin and doxorubicin is in part due to the increase expression of DNA repair enzyme genes, *MLH1* and *MSH2* in sarcoma stem cells [86]. Thus, targeting DNA repair enzymes represent another treatment option in sarcoma stem cells, enhancing the potential DNA damage caused by chemotherapy.

#### 3.1.4. mTOR Pathway

The mTOR pathway has been described to have an important function in CSCs maintenance and survival and drug resistance [87]. Clinical trials in sarcoma with mTOR inhibitors as single agents have shown modest efficacy in STS [88,89]; however, the combination of mTOR inhibitors and conventional chemotherapy has yielded more promising results [90].

In line with this, a Phase I/II clinical trial was conducted with the combination of liposomal doxorubicin and temsirolimus [91], based on pre-clinical evidences in which it was demonstrated that inhibition of mTOR increases the sensitivity of CSCs to doxorubicin [91,92]. In this trial, the combination of liposomal doxorubicin and temsirolimus has led to a PFS three times longer, compared to the results obtained with a mTOR inhibitor in monotherapy [89], and at least two times longer compared with liposomal doxorubicin alone or in combination with other conventional chemotherapy agents [93,94]. It was also shown that the response to treatment correlates with previous laboratory evidence, in which the combination reduced ALDH-high fractions of putative sarcoma stem cells [91]. Hence, mTOR inhibitors cause an increase in the efficacy of chemotherapy, probably by sensitizing chemoresistant CSCs. A phase II trial combining sirolimus with cyclophosphamide in metastatic or unresectable myxoid liposarcoma and chondrosarcoma is currently ongoing (NCT02821507). The association between ALDH expression and mTOR pathway and the recent data suggesting a potential role for ALDH in stemness, highlights also the chance of targeting mTOR pathway/ALDH axis to overcome chemoresistance [95].

#### 3.1.5. Wnt Pathway

Wnt signaling normal function is to regulate the development and homeostasis of many tissues, including those having a mesenchymal origin. Its key role in CSC is well known [13]. Wnt/β-catenin canonical pathway has been involved in MSC differentiation and senescence, as MSC immortalization can be achieved by a Wnt inhibitor, Dickkopf-1 [96,97]. There is recent evidence of a stem cell plasticity that enables cancer cells to dynamically shift between a differentiated and a stem-like state, and changes in Wnt levels are one of the crucial mechanisms in this process [98]. This new role in cell plasticity is a potential cancer therapy new avenue, especially for cells with stemness properties which are drivers of drug resistance. Several drugs that inhibit Wnt signaling could be used to target the CSC niche. This is the case of antibodies targeting LGR5 which have been reported to have therapeutic activity when conjugated to cytotoxic drugs in mouse models of colorectal cancer [99,100]. Tumors carrying inactivating RNF43 mutations depend on paracrine Wnt signaling to activate the stem cell program. Blockade of Wnt secretion by PORCN inhibitors [101] has shown promising therapeutic activity in vitro and are currently being tested in Phase I clinical trials for several solid tumor malignancies. Frizzled Wnt receptors or ligands as RSPO3 can also be targeted with antibodies like vantictumab or rosmantuzumab respectively, which are currently in Phase I clinical trials. At present, just one of these drugs which have entered clinical trials is being tested in sarcomas: Phase I study to address the biodistribution, the safety and recommended dose of a monoclonal antibody that targets Frizzled Homolog 10 in SS. Thus, Wnt signaling inhibition is still in incipient steps for real clinical application, but studies show promising results for the coming years. In the case of STS, increasing evidence suggests that aberrant Wnt signaling can be successfully targeted by Wnt inhibitors in vitro, and with synergistic effects with conventional drugs as doxorubicin or trabectedin [102,103].

#### 3.1.6. ALKATI-Targeted Therapy

As mentioned before, ALKATI is frequently found in STS and it is related with worse survival. From a mechanistic point-of-view, ALKATI was reported to interact with c-Myc, increasing its binding to the ABCG2 promoter and inducing stem cell-like features in STS [104]. ALK inhibitors suppressed stem cell marker expression levels both in vitro and in vivo, therefore probably induced a downregulation of their downstream signaling pathways, and suppressed CSC-like properties [104]. Therefore, ALKATI may be considered a potential prognostic marker and the use of ALK kinase inhibitors a new treatment option for STS patients. Crizotinib, an ALK inhibitor, has being tested in specific subtypes of STS patients (NCT01524926). Of note, ALK inhibitors caused a decrease in stem cell markers expression levels both in vitro and in vivo, that subsequently suppressed the oncogenic functions of ALKATI and attenuated ALKATI-induced CSC-like traits. From a mechanistic point-of-view, ALKATI was reported to interact with c-Myc, increasing its binding to the ABCG2 promoter and inducing stem cell-like features in STS [104].

### 3.2. Targeting the Tumor Microenvironment

The TME is formed by CSCs and MSCs, inflammatory cells, vascular cells and pericytes, the extracellular matrix, and soluble molecules such as chemokines and cytokines, which are secreted by the different cells that constitute the TME. This niche supports the entrance of CSCs in a quiescence state, their stem state and the regulation of their proliferation and differentiation [105]. Besides, the principal component of the TME is the hyaluronan (HA) network, which regulates cell motility and division, and cancer progression and metastasis. Several studies have linked the expression of CD44, one of the HA receptors, with the CSC phenotype, thus converting this protein in a potential therapeutic target [106]. High CD44 expression was associated with worse outcome in STS [32]. Current therapeutic approaches include neutralizing antibody, peptide mimetic, aptamers, natural compounds suppressing CD44 expression, targeting HA by bio-conjugates and nanoparticles, CD44 decoys, and HA oligomers. Studies targeting CD44 are in different preclinical and clinical stages for some cancer types, but are still lacking for STS [107].

On the other side, tumor associated macrophages (TAMs) are core cells of the immune system that determine the intra-tumoral immune status and define cancer cell and immune cell interaction within the TME. TAMs have been described to have two phenotypes: pro-inflammatory M1 macrophages with anti-tumoral functions, and anti-inflammatory M2 macrophages with tumor-supporting properties. Besides, phenotype and function of these monocyte-derived cells can shift between the two extremes. M2 TAMs express CD163, CD115, and CD206; they are able to secrete IL-10, promote tumor growth, and foster angiogenesis. TAMs are crucial for many types of sarcoma but their potential role has been highlighted in leiomyosarcoma [108], and their prognostic value in solitary fibrous tumor [109]. Noteworthy is that TAMs are able to induce the CSCs phenotype and to support CSCs tumorigenic functions, thus representing attractive therapeutic targets. TAMs-depleting agents, or M2 to M1 phenotype converting drugs, are desirable [110]. In line with this, trabectedin, which seems to be capable to eliminate CSCs sub-populations, has been also described to strongly decrease the density of mononuclear phagocytes, including TAMs [111].

Furthermore, CSCs could be partly generated by the mechanism of epithelial–mesenchymal transition (EMT). In cancer cells the EMT induces epigenetic modifications that result in heritable phenotypic changes without causing new genetic alterations. Conversely, a reverse process resembling mesenchymal-to-epithelial transition (MET) occurs in STS, where epithelial differentiation is related to less aggressive cancers and to a favorable prognosis. Given the complexity of EMT/MET processes and the capacity of cancer cells to adapt to stressful conditions, therapeutic strategies affecting one protein or pathway may not be enough to block EMT/MET [112,113,114]. Thus, further preclinical and clinical studies are required to address the mechanisms governing EMT/MET and the potential value of targeting this mechanism in order to eliminate CSCs. A MET transcription profile was correlated with enhanced survival in sarcoma patients [115,116].

In addition, the hallmark of EMT is the loss of the cell adhesion molecule E-cadherin, encoded by *CDH1* gene. In this instance, intercellular junctions and cell–cell interactions are lost, and the intermediate filament composition results altered, allowing cells to dissociate and gain migratory potential. A decrease in E-cadherin expression was associated with poor prognosis in sarcomas [112,113,114]. Moreover, a number of key EMT TFs have been described to regulate E-cadherin expression through direct transcriptional repression of CDH1; among them are SNAIL/SNAI1 and SLUG/SNAI2. Intriguingly, the suppressive effects of SNAI1 or SNAI2 on E-cadherin expression can be prevented through their interaction with the fusion proteins SYT-SSX1 and SYT-SSX2 [112]. Also, SNAI2 has been described to negatively regulate E-cadherin expression in leiomyosarcoma [117]. Hence, SNAI1 and SNAI2 could be potential therapeutic targets in STS. Besides, since miRNAs are involved in the regulation of EMT/MET processes, they can affect the mesenchymal status of sarcomas. A better knowledge of the biology of miRNAs in sarcomas will possibly lead to design novel diagnostic and therapeutic approaches.

### 3.3. Differentiation Therapy

Human leukocyte class I (HLA-I) proteins are crucial in the antigen presentation process to CD8+ cytotoxic T-lymphocytes, and are widely expressed on the cell surface of all human tissues. However, HLA-I-low/negative cells have been reported to escape host immune surveillance due to proliferative advantages, and they may act as TICs [118]. In fact, HLA-I-negative TICs were found in a large variety of sarcoma subtypes, inter alia clear cell sarcoma, leiomyosarcoma, pleomorphic liposarcoma, MPNST and dedifferentiated liposarcoma [119]. These undifferentiated HLA-I-negative cells can generate HLA-I+ cells, which are differentiated cells with lower tumorigenic capacity. Thus, HLA-I expression can be restored by differentiation therapies or certain epigenetic approaches allowing the immune system to recognize CSCs, with the consequently decrease in tumor progression [119].

Another differentiation approach has been described in osteosarcoma. Inhibition of Rho-associated coiled-coil containing protein kinase (ROCK) by fasudil caused terminal adipocyte differentiation in chemotherapy-resistant OS CSCs [120]. This approach takes advantage of the pluripotency of CSCs to induce their trans-differentiation into another lineage; however, its therapeutic value is still unknown in STS.

### 3.4. Directed Immunotherapy

In addition to the differentiation therapy, cytokine-induced killer (CIK) cells that constitute a HLA-I-independent tumor cell killing may be another promising approach for HLA-I-negative cells targeting [121]. CIKs are derived from T lymphocytes ex vivo expansion and show a T-NK phenotype and MHC-independent antitumor ability. Studies demonstrated, first in pre-clinic models and then in the clinical context, that autologous CIK cells were active against CSCs that survived treatment with doxorubicin or pazopanib [122]. On the other hand, CIK cell-mediated immunotherapy can be contemplated synergistically with other immunotherapies, such as T cell checkpoint inhibitors, for example cytotoxic T-lymphocyte-associated antigen 4 (CTLA-4), programmed cell death-1 (PD-1) or its ligand (PDL-1). In fact, preclinical data suggest that combined therapy may be synergistic as CIK cells express PD-1 on their membrane [123,124]. The activity of this combination against CSCs should be addressed in future studies in STS.

### 3.5. Epigenetic Modulation

Epigenetic modifiers, as mentioned before, seem to have an important role in sustaining a stem-like phenotype in STS. As an example, high expression of LIN28B has been associated with doxorubicin resistance and worse survival, pointing that this protein may be a relevant therapeutic target in STS [66]. Accordingly, the development of a new LIN28B-targeted therapy, including immunotherapy, might be a good approach in STS.

On the other hand, histone deacetylase inhibitors (HDACi) are epigenetic modifiers able to inhibit sarcoma growth, induce tumor cell apoptosis and arrest cell cycle, impair tumor invasion, metastasis and angiogenesis, regulate autophagy, generate reactive oxygen species (ROS) and induce cell differentiation. Importantly, novel HDACi have been proposed to counteract sarcoma CSCs. These compounds not only sensitized sarcoma cells to chemotherapy by reversing multidrug resistance, but also increased treatment responses when in combination with chemotherapy [125,126,127,128]. In a phase I/II clinical study evaluating the HDACi belinostat in combination with doxorubicin, in a cohort of 41 STS patients, the response rate was modest but median PFS was 6 months. A figure described to be superior to some other reports using doxorubicin as single-agent [129]. Moreover, recent preclinical studies indicated that HDACi can raise NK cell action against sarcoma cells, and that HDACi combined with PI3K/mTOR inhibitor, DNMT inhibitor or proteasome inhibitor may also represent a successful treatment option for sarcoma patients. These combinations seem more promising than HDACi in monotherapy [125,126,127,128].

Likewise, EZH2-targeted therapy against CSCs could be proposed, since its overexpression has been detected in several tumors, including sarcomas. EZH2 mediates H3K27me3 silencing of tumor suppressor genes, enabling CSC expansion [17]. Recently, the new and first-in-human EZH2 inhibitor, tazemetostat, showed an encouraging anti-tumor activity in SMARCB1-deleted sarcomas [130]. In addition, it has been shown that EZH2 inhibition is able to suppress leiomyosarcoma CSCs proprieties and retrieve the anti-tumor effect of PI3K/mTOR inhibition, supporting the therapeutic value of this combination [131]. As mentioned, altered expression of EZH2 in ERMS and SS has been linked to cancer survival and to retention of a less differentiated and more aggressive phenotype. Thus, EZH2 pharmacological inhibition could be exploited as a differentiation therapy in STS [69,71,72]. Tazemetostat, has been shown to be active in epithelioid sarcoma and it is the first FDA-approved targeted-therapy for the treatment of this rare STS subtype [132,133].

## 4. Conclusions

Overall, the rarity and the heterogeneity of sarcomas turn the development of new therapeutic options in a complete challenge.

There is clear evidence that proves the existence of CSCs in many cancer types, but our understanding of the cell hierarchies present in tumors is still lacking. Future directions include the definition of CSC hierarchies and tracking of the origin of CSCs, which will identify critical therapeutic targets to improve patient outcomes and design more effective and personalized therapeutic approaches. For that, the use of lineage tracing, single cell analysis and 3D culture systems is fundamental [134].

In vivo, the presence of CSCs with a slow-cycling status hinders their targeting, as these cells will resist anti-proliferative molecules found out through screening approaches on proliferating CSCs in vitro. Thus, models of quiescent CSCs have to be developed to identify potentially successful strategies. In addition, new technologies have to be implemented to analyze CSCs in their intact environment to know how the tumor niche affects the CSC status.

Another critical issue is plasticity, since there is a transformation between stem cells to non-stem cells or vice versa. Plasticity is a feature of the TME, which leads to tumor aggressive behavior and to the development of phenotypically unstable heterogeneous cell populations within the same tumor or between tumors from different patients [1]. Thus, the existence of a “one-size-fits-all” CSC-targeted therapy or a single drug that offers better clinical results in all sarcomas subtypes is unlikely. Accordingly, the goal of future research must be the development of combination therapies based in the genetic/epigenetic alterations, addressed to each histologic subtype and to each patient response, together with the identification and understanding of the mechanisms involved in the reversion that may occur after the elimination of the CSC population.

Nevertheless, the principal barrier to target CSCs seems to be toxicity. Most of the therapies are directed against biomarkers expressed by CSCs but also by normal stem cells. In fact, recent clinical trials have shown several therapies against CSCs with high side effects [135]. Therefore, future studies should be oriented to define new and reliable methods to isolate and characterize sarcoma CSC populations, as well as to identify novel CSCs-directed therapies with a low toxicity profile.

In addition, EMT is usually related to CSC features such as tumor-propagating potential. Further research is needed to determine if EMT is a common feature of CSCs or if EMT cells act as a different group of CSCs with more slow-growing status and invasive properties, leading to metastasis, whereas the majority of tumors are maintained by multipotent CSCs that do not show EMT features [136]. Accordingly, another question to be addressed is how the intermediate states of CSCs during EMT are controlled and what their effects are.

Until now, no research has efficiently overcome CSC chemotherapy resistance. Discussions about CSCs origin persist and we still lack an accurate comprehension of their role in cancer initiation and progression. Although we are beginning to observe important clinical successes that increase hope that CSC-targeting drugs have an encouraging future, the specificity of the targeted antigens remains a challenge.

The future objective is to define the populations of sarcoma stem cells and identify novel therapeutic agents to target these cells. Since signature translocations that drive tumorigenesis have already been identified in many STSs, and since CSCs express stemness markers, to look for a relation between both could be useful for therapy.

Large scale analysis of tumor samples using genomics, proteomics and ‘big data’ could open up new opportunities for the identification of CSC-specific markers for each sarcoma subtype that will allow the development of more efficient CSC targeted therapies. In addition, these approximations could also lead to personalized therapies and reduced costs and toxicity.

In conclusion, independent of the therapy approach used, the specificity of the targets is key for an efficient elimination of CSCs and cancer recurrence.

## Figures and Tables

**Table 1:** Overview of Cancer stem cell (CSC)-associated markers and features in some soft-tissue sarcoma (STS) subtypes.

STS Subtype	CSC Marker/Feature	Evidence
Clear Cell Sarcoma	EZH2 [73]	Patient samples
Fibrosarcoma	ALDH [40]	In vitro
	CD133 [22]	In vitro
	PDGFRα and PDGFRβ [44]	In vitro and in vivo
	Embryonic stem cell transcription factors [24]	In vitro and in vivo
Leiomyosarcoma	PDGFRα and PDGFRβ [44]	In vitro and in vivo
Liposarcoma	ALDH [41]	In vivo and patient samples
	CD133 [41]	In vivo and patient samples
	DNMT1 [67]	In vitro and in vivo
	PDGFRα and PDGFRβ [44]	In vitro and in vivo
Myxoid Liposarcoma	SWI/ SNF, ISWI and CHD [74]	In vitro
MPNST ^1^	Nestin [36]	In vitro and in vivo
Rhabdomyosarcoma	ALDH [39]	In vitro and in vivo
	CD133 [19,20,21]	In vitro, in vivo and patient samples
	DNMT3B [68]	In vitro
	EZH2 [69]	In vitro and in vivo
	Nestin [19,27,33,34,35]	In vitro, in vivo and patient samples
	NOTCH-YAP1-Sox2 [62]	In vitro, in vivo and patient samples
	Side Population [54]	In vitro, in vivo and patient samples
	Embryonic stem cell transcription factors [64]	In vitro, in vivo and patient samples
	SWI/ SNF, ISWI and CHD [76]	In vitro, in vivo and patient samples
Synovial Sarcoma	ALDH [40]	In vitro
	Active Wnt pathway [77]	In vitro and in vivo
	BMI1 [23]	In vitro
	CD133 [23,25]	In vitro and patient samples
	CXCR4 [28]	In vitro, in vivo and patient samples
	EZH2 [70,71,72]	In vitro, in vivo and patient samples
	SWI/ SNF, ISWI and CHD [75]	Patient samples
UPS ^2^	Side Population [54]	In vitro, in vivo and patient samples
	ABC transporters [51]	In vitro
	CD133 [51]	In vitro
	Embryonic stem cell transcription factors [51]	In vitro

^1^ MPNST: Malignant peripheral nerve sheath tumor; ^2^ UPS: Undifferentiated pleomorphic sarcoma.
